# A Protest from the I. H. A.

**Published:** 1895-01

**Authors:** B. L. B. Baylies, J. H. Allen

**Affiliations:** President; Vice-President


### A PROTEST FROM THE I. H. A.

EDITOR OF THE HOMŒOPATHIC PHYSICIAN:

The statement made by the author of the preamble to the announcement of the nascent society, the "New Society of Homœopathicians," published in the November number of THE HOMŒOPATHIC PHYSICIAN, that the I. H. A., by the indefinite postponement of the report of its Board of Censors, June 21st, 1892, "*deliberately violated* its Declarations of Principles, Constitution, and By-laws," can hardly be otherwise than false; for the author assumes to be informed, and if a member was doubtless informed of the following facts: Charges had been made against a member of that body of certain errors of practice conflicting with its laws and principles; that report upon these charges would be made by the Censors was generally believed; but to some extent the impression prevailed that the interests of the association would best be served by omitting their consideration; as the member had already presented his defense in a circular letter embracing the particulars charged, and had obtained the opinions of many individuals upon the question of his culpability or error. But the "violation" involved in the indefinite postponement of the report at that meeting was not deliberate; the motion to postpone was suddenly and unexpectedly sprung upon the assemblage, and without deliberation as to its actual character and effects, its bearing upon the laws and principles of the I. H. A., without opportunity for discussion or debate, possibly under a bias of judgment produced by the accused member's circular, was passed with impulsive acclamation. One or more of the members of the "new society" voted with the majority. The "immortal thirteen," most of whom have, we believe, entered this new organization, not surprised into precipitancy by the *coup d'état*, voted in the negative; whether actuated by prior knowledge, counter bias, or superior and more deliberate judgment.

Why did not this *fortunate* minority object to the deplored proceeding, debate the question, and prevent the vote? They protested by a negative; but too late.

A certain amount of wisdom remained with the majority. They regretted this precipitate action and the default of justice, but well knew the *temple had not been destroyed.* A breach had been made, which could be repaired, and its integrity reassured. They painfully reflected upon their unwitting disrespect to the Chairman of the Board, whom all affectionately esteem, and for which they immediately strove to atone. They hoped also that the members who have seen fit to withdraw from the parent body would not sever their connection with it; who even now, must realize that the abusive and false sentiment expressed in the preamble announcement of the "New Society of Homœopathicians" can be entertained by none but its unknown author, and must hope that it will not recoil injuriously upon him or his fellows.

There cannot be too many societies engaged in the study of Hahnemann's Therapeutic Philosophy and the beneficent practice of his precepts: the more, so much the better for physicians, and best for the world.

Let us live in harmony, for we run the same race and aspire to the same goal.

In June of the present year, at Niagara, the I. H. A. unanimously adopted the following resolution presented by its Board of Censors:

"Many members recall with regret the passage of a resolution by our Association at Narragansett Pier, June 21st, 1892, which indefinitely postponed a report then offered by the Board of Censors, thus thwarting their performance of a mandatory duty.

"With the hope to repair as far as possible its results, the Board of Censors presents the following:

"'Whereas, It is claimed by some of our highly esteemed colleagues that at the meeting held by our Association at Narragansett Pier, June 21st, 1892, its By-Laws were infringed and its principles imperilled by the hasty passage of a resolution to indefinitely postpone a report of the Board of Censors, this Association hereby renounces such action as a precedent, and reaffirms its adherence to its Constitution, By-Laws, and principles. All of which was respectfully submitted and unanimously adopted.'"

Since the event of 1892, the I. H. A., as in all previous years of its existence, has maintained a healthy, well-ordered organization, its regular annual meetings, and performed its work with the accustomed emulation of its members, for purity of practice and devotion to its principles worthily recorded in its transactions.

B. L. B. Baylies, M. D.,
*President.*

J. H. Allen, M. D.,
*Vice-President.*

